# 2,2′-(Ethane-1,2-di­yl)bis­[3-(pyridin-2-yl)imidazo[1,5-*a*]pyridin-2-ium] dichloride hexa­hydrate

**DOI:** 10.1107/S2056989026009229

**Published:** 2026-09-11

**Authors:** Olga Yu. Vassilyeva, Vladimir N. Kokozay, Evgeny Goreshnik

**Affiliations:** a Department of Chemistry, Taras Shevchenko National University of Kyiv, 12 Hetman Pavlo Skoropadskyi St., 01033 Kyiv, Ukraine; b Department of Inorganic Chemistry and Technology Jozef Stefan, Institute, Jamova 39 1000 Ljubljana Slovenia; Texas A & M University, USA

**Keywords:** crystal structure, twin, ethyl­enedi­amine, 2-pyridine­carbaldehyde, cyclo­condensation

## Abstract

The title compound, C_26_H_22_N_6_^2+^·2Cl^−^·6H_2_O, crystallizes in the monoclinic space group *P*2_1_/*n* with one mol­ecule in the asymmetric unit. Structure determination at 150 K from a two-component twin crystal reveals the non-symmetrical n-shaped dication, where two pyridine–imidazole fused cores bearing pendant pyridyl rings are linked by an ethyl­ene bridge. The fused five- and six-membered rings are almost coplanar with the largest deviation from the mean plane being 0.019 Å for a nitro­gen atom of one of the rings. The pendant pyridyl groups are twisted by 42.14 (11) and 46.47 (11)° with respect to the planes of the adjacent imidazo[1,5-*a*]pyridinium cores. In the crystal, the dications stacked along the *b-*axis direction are linked by π–π inter­actions that possibly stabilize the n-type structure in the solid state. The chloride anions and water mol­ecules form *R*^2^_4_(8), *R*^5^_5_(12), *R*^6^_6_(12) and *R*^8^_11_(22) ring motifs through O—H⋯O/Cl hydrogen bonds, building corrugated layers that run between columns of the stacked organic dications.

## Abstract 2

The title compound crystallizes as a two-component twin in space group *P*2_1_/*n* with *Z′* = 1, exhibiting hydrogen-bonding corrugated layers of chloride ions and water mol­ecules that run approximately along the diagonal plane of the cell between columns of the stacked dications.

## 1. Chemical context

Versatile optoelectronic properties, structural richness, and low temperature solution processability make organic–inorganic hybrid compounds based on metal halides an appealing alternative to conventional photovoltaic devices that utilize crystalline silicon solar cells (Zhu *et al.*, 2022 ▸). Hybrid halometalates have been also actively researched for other applications as promising materials for light-emitting diodes (LEDs), semiconductor optical amplifiers, X-ray detectors, and lasers (Arya *et al.*, 2024 ▸; You *et al.*, 2023 ▸). The choice of appropriate organic cation is crucial to the structural consolidation and performance of the organic–inorganic hybrid system (Kucheriv *et al.*, 2025 ▸).

Recently, we have developed a convenient synthetic technique to prepare hybrid salts consisting of functionalized imidazo[1,5-*a*]pyridinium cations and halometalate anions (Vassilyeva *et al.*, 2020 ▸, 2023 ▸). The fused nitro­gen-containing bicyclic systems, imidazo[1,5-*a*]pyridines, are valuable in many research areas including materials science and pharmaceuticals (Esken *et al.*, 2025 ▸; Gopathi *et al.*, 2025 ▸). They exhibit intense fluorescence and high quantum yield (Volpi, 2022 ▸; Vasylets *et al.*, 2026 ▸). In the developed synthetic procedure, the cation is produced in the inter­action between equimolar amounts of amine, formaldehyde (FA) and 2-pyridine­carbaldehyde (2-PCA) in aqueous solution and is used without isolation (Vassilyeva *et al.*, 2020 ▸, 2021 ▸, 2023 ▸). The cyclo­condensation is catalysed by acid introduced as an adduct of the amine. The preformed cations synthesized with use of methyl­amine or ethano­lamine hydro­chlorides easily formed organic–inorganic hybrid compounds with metal halides (*M* = Mn, Co, Fe, Ni, Cu, Zn, Cd, Pb, Sn; Hal = Cl, Br, I). A similar outcome was expected for PdCl_2_, which willingly produces organic–inorganic hybrid salts based on the square-planar [PdCl_4_]^2–^ anion (Salah *et al.*, 2023 ▸). The replacement of the amine with ethyl­enedi­amine (En) in the reaction with PdCl_2_ using the molar ratio 2-PCA:FA:En·2HCl = 4:4:1 led to the isolation of the unexpected organic chloride salt with a novel imidazo[1,5-*a*]pyridinium-based dication as a minor product, whose identity was established crystallographically. We can speculate that the intrinsic catalytic activity of Pd^2+^ cations was responsible for its formation. Herein, we report the synthesis and crystal structure of [*L*]Cl_2_·6H_2_O (I), where [*L*]^2+^is 2,2′-(ethane-1,2-di­yl)bis­(3-(pyridin-2-yl)imidazo[1,5-*a*]pyridin-2-ium) dication, which crystallizes as a two-component twin.
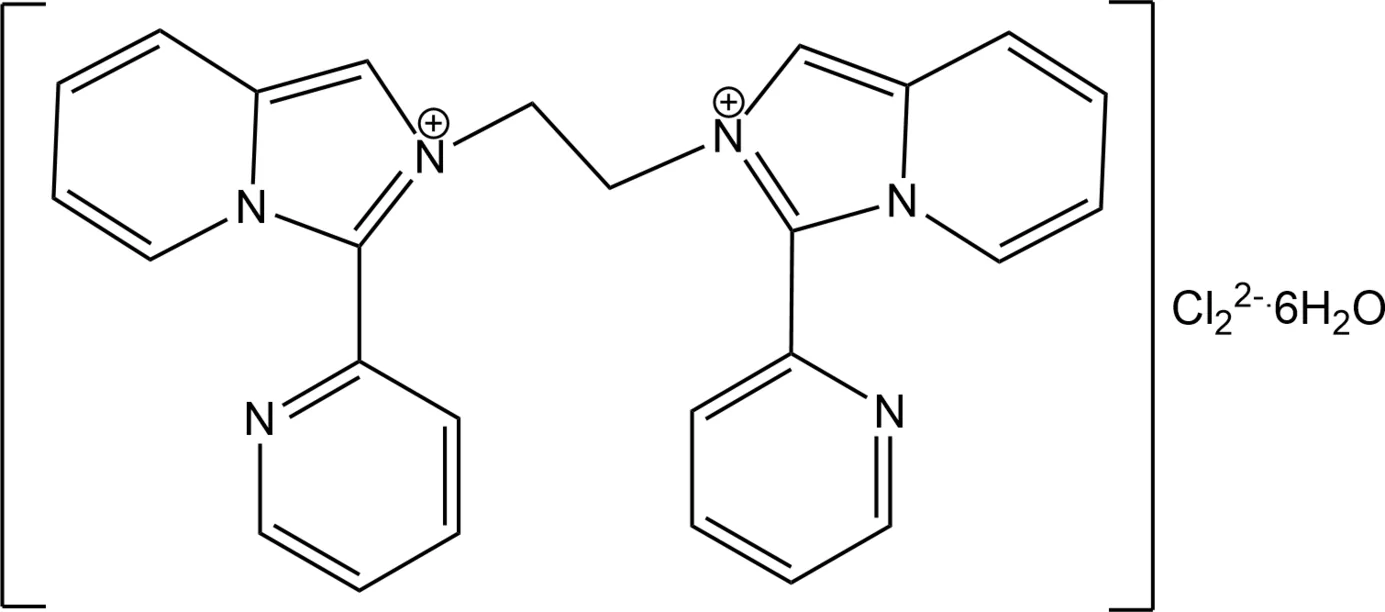


## 2. Structural commentary

**Figure 1:**
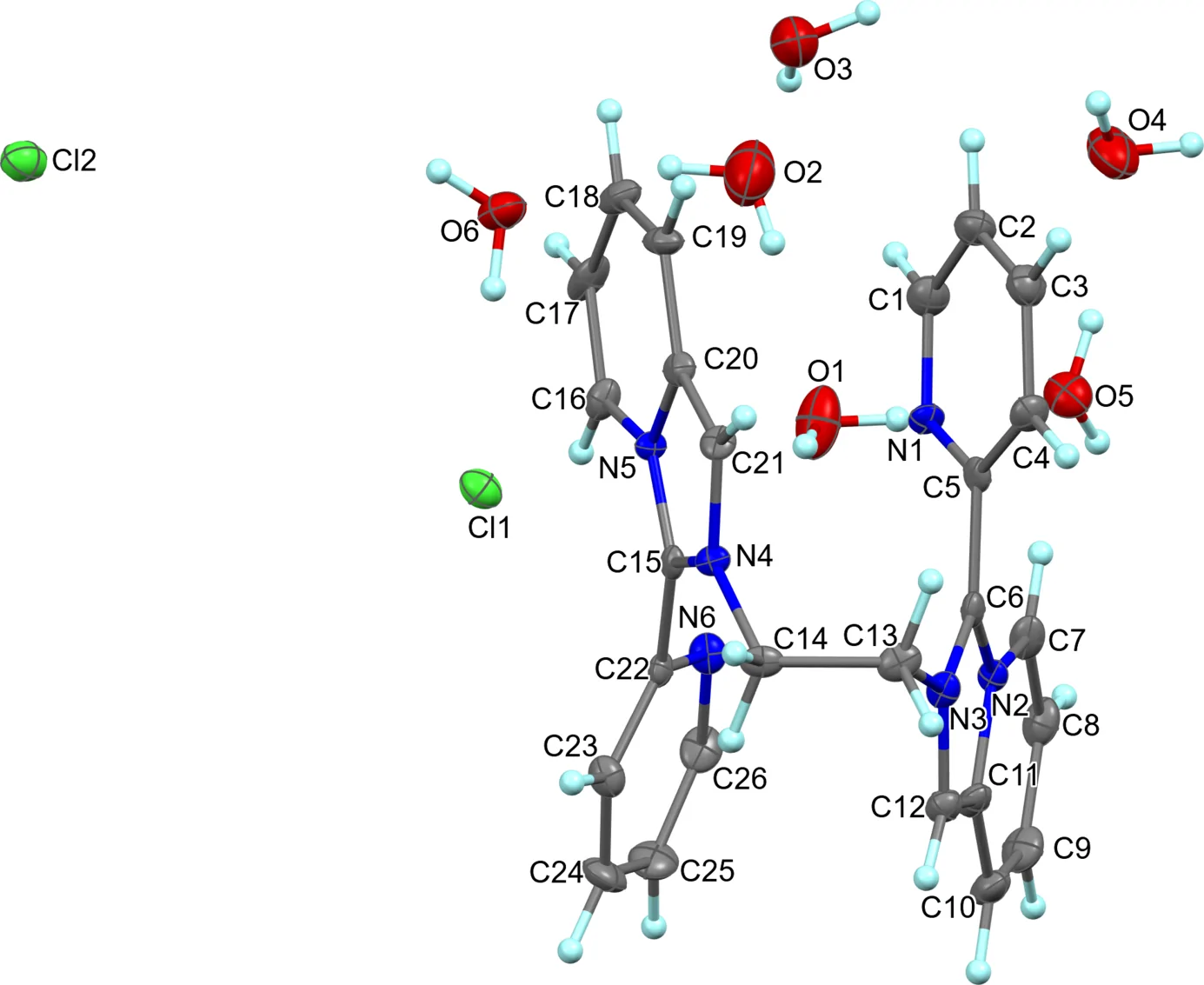
Mol­ecular structure of the title mol­ecular salt, with the atom labelling and displacement ellipsoids drawn at the 50% probability level.

Monoclinic crystals of [*L*]Cl_2_·6H_2_O, which crystallize in the space group *P*2_1_/*n*, contain discrete organic cations, chloride anions and water mol­ecules of crystallization (Fig. 1 ▸). In the n-shaped dication, two pyridine–imidazole fused cores bearing pendant pyridyl rings are linked by an ethyl­ene bridge. The pyridinium rings in the flattened fused moieties have expected bond distances; the bond lengths in the imidazolium entities fall in the range 1.342 (3)–1.407 (3) Å (Table 1 ▸). The fused five- and six-membered rings are almost coplanar, with the largest deviation from the mean plane being 0.019 Å (N5). The pendant pyridyl rings are twisted by 42.14 (11) (N1) and 46.47 (11)° (N6) with respect to the planes of the adjacent imidazo[1,5-*a*]pyridinium cores. The geometric parameters of the 3-(pyridin-2-yl)-imidazo[1,5-*a*]pyridinium moieties in the dication are similar to those found in related structures [Cambridge Structural Database (CSD) refcodes HUMCUP (Buvaylo *et al.*, 2015 ▸) and GOSYUL (Vassilyeva *et al.*, 2021 ▸)].

## 3. Supra­molecular features

**Figure 2:**
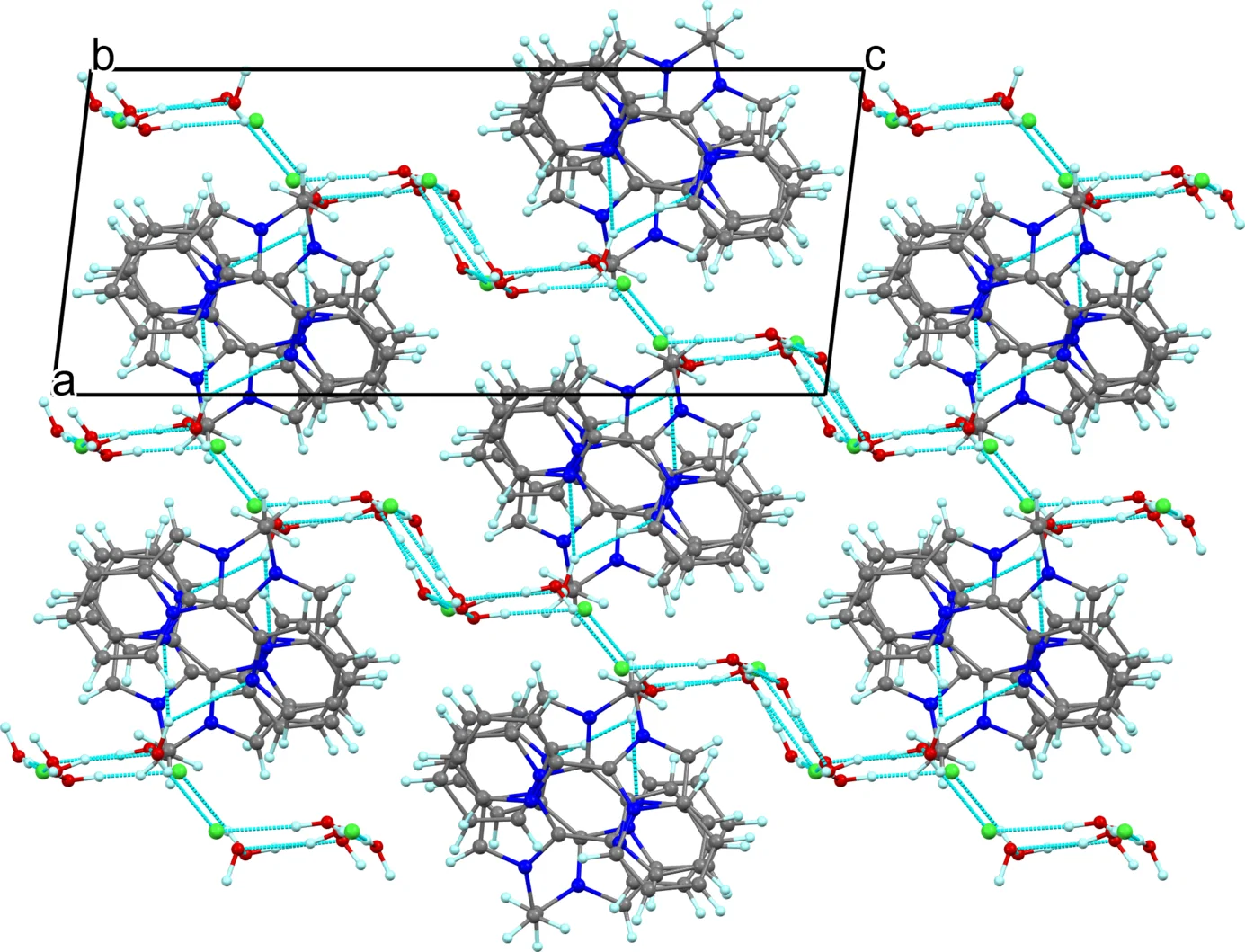
Crystal packing of (I), viewed along the *b* axis, showing stacks of organic dications between hydrogen-bonding corrugated layers formed by chloride anions and water mol­ecules. Hydrogen bonds are shown in blue.

**Figure 3:**
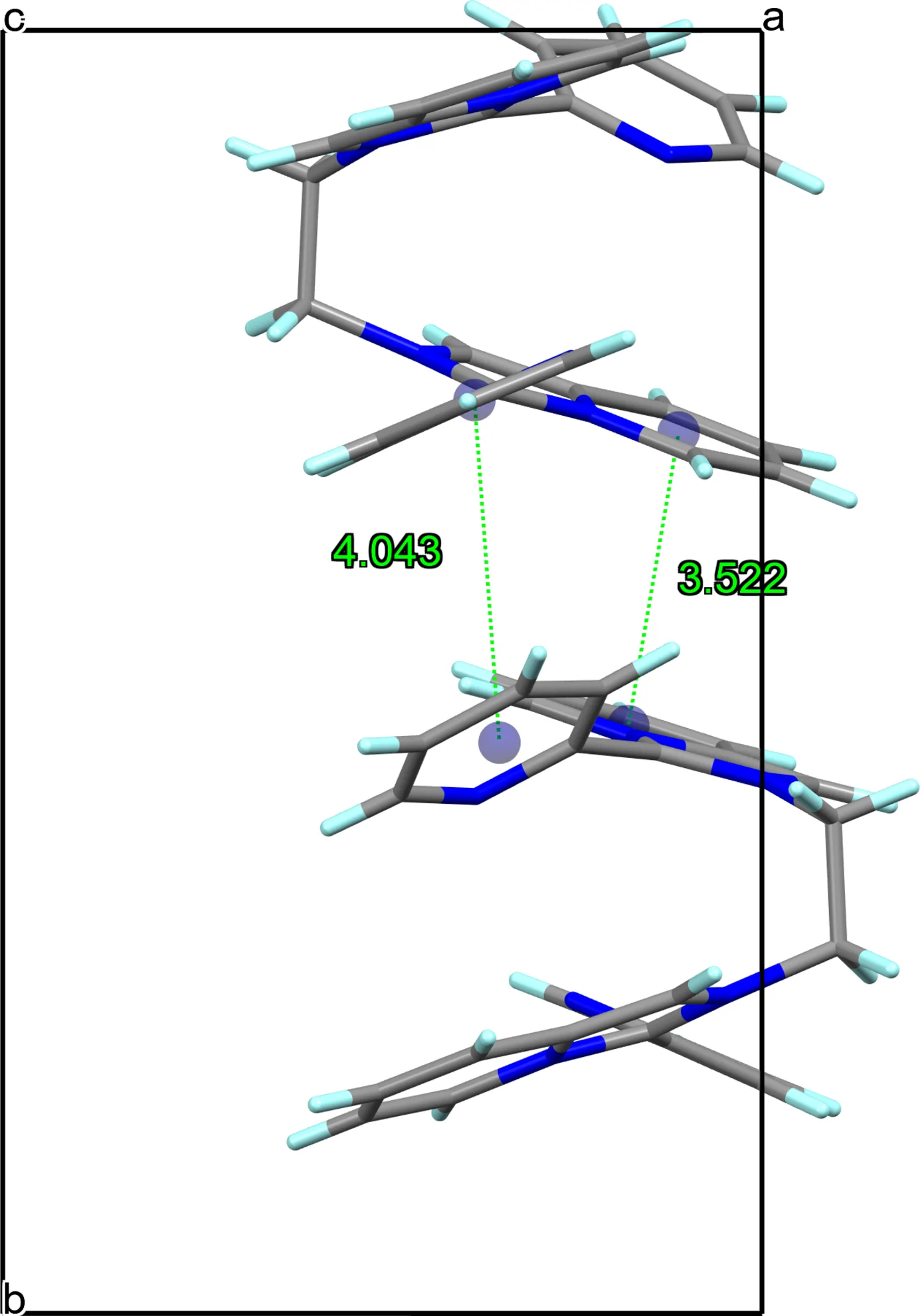
Aromatic stacking of the dications in (I), viewed along the *c* axis.

In the crystal, the dications are arranged in stacks aligned along the *b-*axis direction (Fig. 2 ▸). In a stack, *L*^2+^ cations are disposed in an anti­parallel fashion being linked through two types of stacking contacts. Those involve π–π inter­actions between pendant pyridyl groups and between the six-membered rings of the fused cores from adjacent organic moieties with the centroid⋯centroid distances of 4.043 (2) and 3.522 (2) Å, respectively (Fig. 3 ▸). The aromatic stacking is possibly important in the stabilization of the n-type structure of the dication in the solid state.

**Figure 5:**
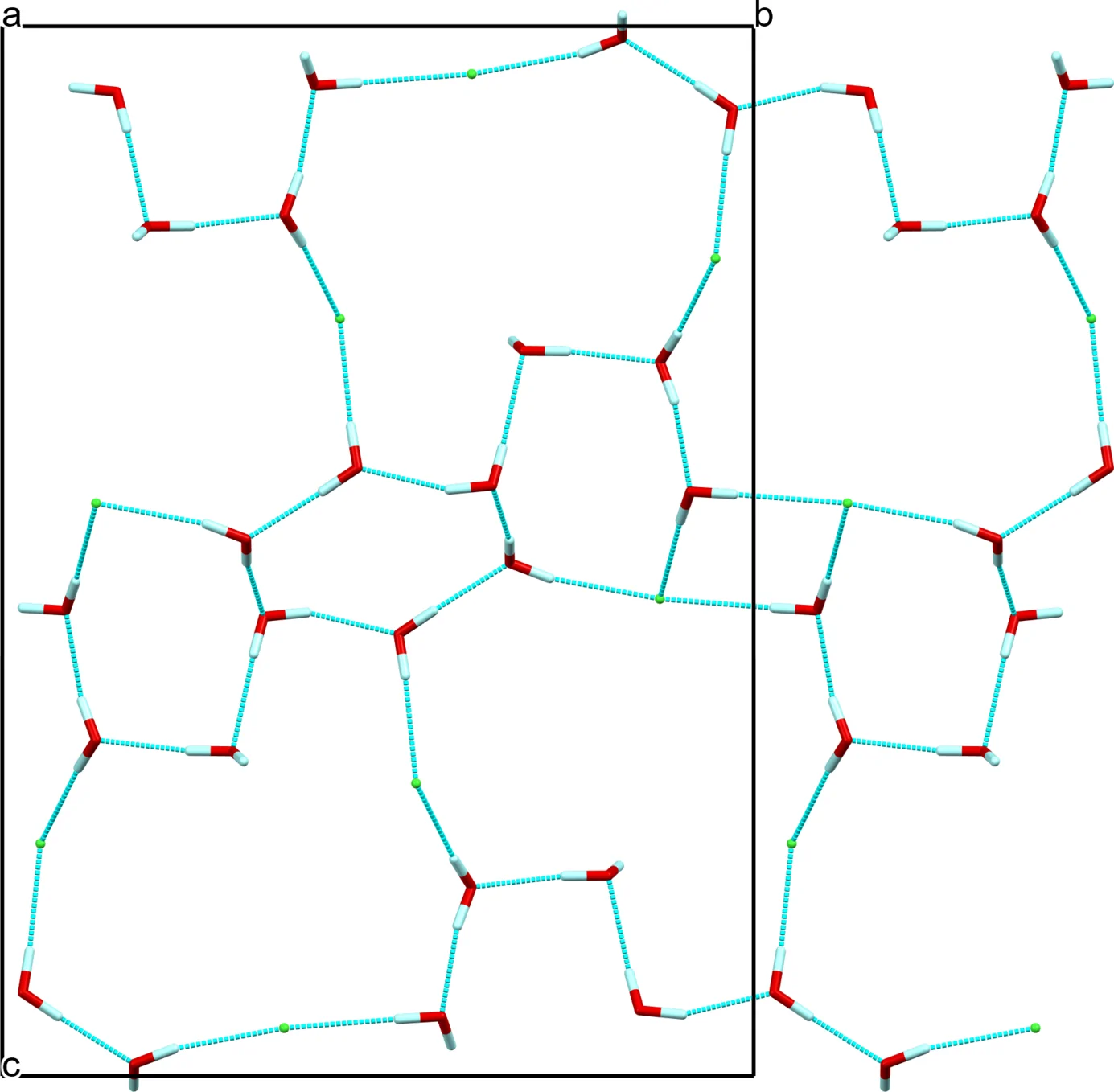
View of the 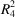
(8), 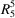
(12), 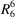
(12) and 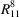
(22) graph-set motifs forming closed rings through O—H⋯O/Cl hydrogen bonds between chloride anions and water mol­ecules in (I).

The chloride ions and water mol­ecules are involved in the O—H⋯O/Cl inter­actions (Table 2 ▸), forming a hydrogen-bonding corrugated layer that runs approximately along the diagonal plane of the monoclinic cell containing the *b* axis, between columns of the stacked dications (Figs. 2 ▸ and 4 ▸). Using graph-set notation, there are several different hydrogen-bonding ring motifs: 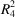
(8) (two water mol­ecules and two chlorides), 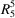
(12) (five waters and a chloride), 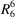
(12) (six waters), 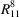
(22) (eight waters and three chlorides) (Fig. 5 ▸).

## 4. Database survey

120 structures of compounds featuring the 3-(pyridin-2-yl)imidazo[1,5-*a*]pyridine fragment are found in the CSD (Version 5.45, update Mar 2026; Groom *et al.*, 2016 ▸) including the structure of neutral 3-(pyridin-2-yl)imidazo[1,5-*a*]pyridine itself (PRIMPY; Golič *et al.*, 1980 ▸). Unlike compound (I), the mol­ecule of the latter as a whole is planar with the largest deviation from the mean plane of 0.06 Å for the pendant pyridyl group N atom (PRIMPY02; Tian *et al.*, 2026 ▸). PRIMPY and its derivatives as ligands create a favourable coordination environment for cation binding through the pyridyl and imidazo ring nitro­gens giving rise to a variety of metal complexes. For example, in chloro-bis­(3-(pyridin-2-yl)imidazo[1,5-*a*]pyridine)­copper(II) chloride ethanol solvate (ELILOD; Carson *et al.*, 2021 ▸), the ligands retain planarity upon coordination.

Functionalization of 3-(pyridin-2-yl)imidazo[1,5-*a*]pyridine at the imidazo ring nitro­gen turns the neutral mol­ecule into organic cation thus encouraging formation of organic–inorganic halometalate salts. 14 crystal structures from our research span hybrid halometalates, metal complexes (LOJFUO and HOMZER, respectively; Vassilyeva *et al.*, 2019*a* ▸) and organic salts (HOMZER; Vassilyeva *et al.*, 2019*b* ▸) with the monopositive 2-methyl-3-(pyridin-2-yl)imidazo[1,5-*a*]pyridin-2-ium cation, which can be seen as half of *L*^2+^. The structural configurations of the monopositive cation with a flattened fused core are similar to that of *L*^2+^ with the degree of twist between the pendant pyridyl rings and the planes of the remainder of the cation as large as 85.30 (3)° (HUMCUP; Buvaylo *et al.*, 2015 ▸).

## 5. Synthesis and crystallization

Formaldehyde solution was prepared by dissolving paraform (0.13 g, 4.5 mmol) in 10 ml boiling deionized water in a 50 ml conical flask. After cooling at r.t., solid En·2HCl (0.26 g, 1.2 mmol) was added to the FA solution, which was stirred at r.t. for half an hour and then filtered. In a week, 2-PCA (0.38 ml, 4 mmol) was introduced into the flask followed by subsequent dropwise addition of PdCl_2_ (0.35 g, 2 mmol) dissolved in CH_3_CN (5 ml). The brownish solution was kept stirring for 1 h, then filtered and left open to stand at r.t. The inseparable mixture of a dark residue and light yellow crystals precipitated over several weeks. The precipitate was filtered off, washed with ^*i*^PrOH and finally dried in air. Yellow needles of (I) suitable for X-ray crystallography that constituted a minor product were withdrawn manually.

## 6. Refinement

Crystal data, data collection and structure refinement details are summarized in Table 3 ▸. The structure was refined as a two-component twin with a ratio of 0.7078 (12):0.2922 (12). Component 2 is rotated by −179.9853° around [0.71 −0.00 −0.71] (reciprocal) or [0.99 0.00 −0.14] (direct). H atoms of the water mol­ecules were located in a difference-Fourier map and refined as free rotating groups. Anisotropic displacement parameters were employed for the non-hydrogen atoms. The H atoms attached to carbon atoms were added at calculated positions and refined as riding with isotropic displacement parameters based on those of the parent atom [C—H = 0.95 Å, *U*_iso_(H) = 1.2*U*_eq_(C) for CH; C—H = 0.99 Å, *U*_iso_(H) = 1.5*U*_eq_(C) for CH_2_].

## Supplementary Material

Crystal structure: contains datablock(s) I. DOI: 10.1107/S2056989026009229/jy2076sup1.cif

Structure factors: contains datablock(s) I. DOI: 10.1107/S2056989026009229/jy2076Isup2.hkl

Supporting information file. DOI: 10.1107/S2056989026009229/jy2076Isup3.cml

CCDC reference: 2585211

Additional supporting information:  crystallographic information; 3D view; checkCIF report

## Figures and Tables

**Figure 4:**
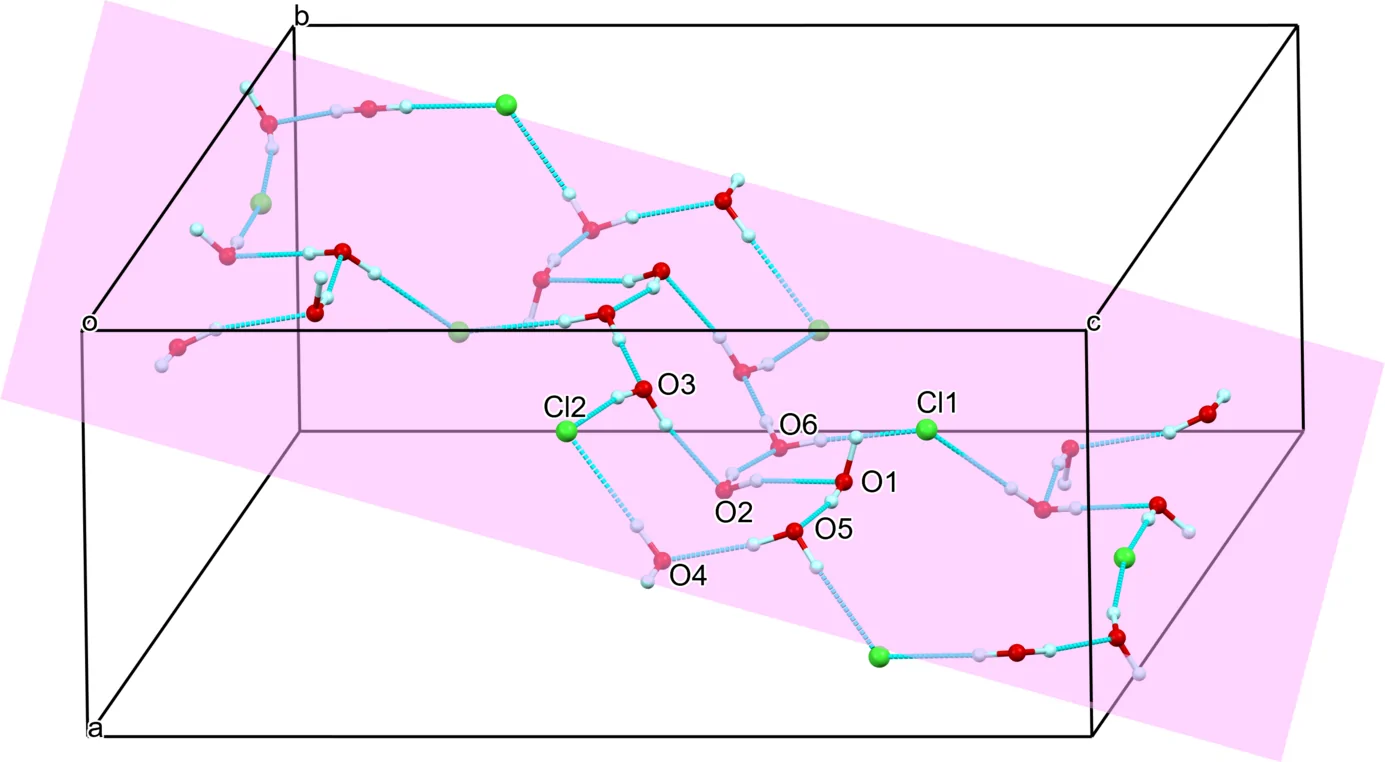
Fragment of the hydrogen-bonding layer in (I), involving chloride anions and water mol­ecules and propagating approximately along the diagonal plane of the monoclinic cell containing the *b* axis (see Table 2 ▸ for details).

**Table 1:** Selected bond lengths (Å)

N1—C1	1.342 (4)	C4—C5	1.378 (4)
N1—C5	1.352 (3)	C5—C6	1.477 (4)
N2—C6	1.342 (3)	C7—C8	1.340 (4)
N2—C7	1.392 (3)	C8—C9	1.423 (4)
N2—C11	1.407 (3)	C9—C10	1.355 (4)
N3—C6	1.351 (3)	C10—C11	1.411 (4)
N3—C12	1.361 (3)	C11—C12	1.360 (4)
N3—C13	1.479 (3)	C13—C14	1.505 (4)
N4—C14	1.464 (3)	C15—C22	1.475 (4)
N4—C15	1.352 (3)	C16—C17	1.340 (4)
N4—C21	1.364 (3)	C17—C18	1.425 (4)
N5—C15	1.345 (3)	C18—C19	1.342 (4)
N5—C16	1.395 (3)	C19—C20	1.416 (4)
N5—C20	1.397 (3)	C20—C21	1.363 (4)
N6—C22	1.346 (3)	C22—C23	1.377 (4)
N6—C26	1.334 (3)	C23—C24	1.382 (4)
C1—C2	1.380 (4)	C24—C25	1.368 (4)
C2—C3	1.366 (4)	C25—C26	1.372 (4)
C3—C4	1.396 (4)		

**Table 2:** Hydrogen-bond geometry (Å, °)

*D*—H⋯*A*	*D*—H	H⋯*A*	*D*⋯*A*	*D*—H⋯*A*
O1—H1*A*⋯O5	0.87	1.89	2.754 (3)	170
O2—H2*A*⋯O1	0.87	2.04	2.891 (4)	164
O2—H2*B*⋯O6	0.87	1.90	2.739 (3)	161
O3—H3*A*⋯Cl2^i^	0.87	2.26	3.119 (2)	169
O3—H3*B*⋯O2	0.87	1.88	2.737 (4)	169
O5—H5*A*⋯O4	0.87	1.90	2.758 (3)	169
O5—H5*B*⋯Cl1^ii^	0.87	2.25	3.117 (2)	176
O6—H6*A*⋯O3^i^	0.87	1.84	2.705 (3)	175
O6—H6*B*⋯Cl1	0.87	2.21	3.081 (2)	175

Symmetry codes: (i) 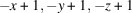; (ii) 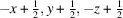.

**Table 3:** Experimental details

Crystal data	
Chemical formula	C_26_H_22_N_6_^2+^·2(Cl^−^)·6(H_2_O)
*M*_r_	597.49
Crystal system, space group	Monoclinic, *P*2_1_/*n*
Temperature (K)	150
*a*, *b*, *c* (Å)	9.0061 (7), 15.1053 (10), 21.2487 (14)
β (°)	96.752 (7)
*V* (Å^3^)	2870.6 (3)
*Z*	4
Radiation type	Mo *K*α
μ (mm^−1^)	0.28
Crystal size (mm)	0.38 × 0.09 × 0.05

Data collection	
Diffractometer	New Gemini, Dual, Cu at home/near, Atlas
Absorption correction	Analytical (*CrysAlis PRO*; Rigaku OD, 2026 ▸)
*T*_min_, *T*_max_	0.933, 0.980
No. of measured, independent and observed [*I* > 2σ(*I*)] reflections	6542, 6542, 3396
*R*_int_	?
(sin θ/λ)_max_ (Å^−1^)	0.678

Refinement	
*R*[*F*^2^ > 2σ(*F*^2^)], *wR*(*F*^2^), *S*	0.051, 0.091, 0.86
No. of reflections	6542
No. of parameters	380
H-atom treatment	H-atom parameters constrained
Δρ_max_, Δρ_min_ (e Å^−3^)	0.27, −0.34

Computer programs: *CrysAlis PRO* (Rigaku OD, 2026 ▸), *SHELXT* (Sheldrick, 2015*a* ▸), *SHELXL2016/6* (Sheldrick, 2015*b* ▸), *OLEX2* (Dolomanov *et al.*, 2009 ▸) and *Mercury* (Macrae *et al.*, 2020 ▸).

## supplementary crystallographic information

### 2,2'-(Ethane-1,2-diyl)bis[3-(pyridin-2-yl)imidazo[1,5-a]pyridin-2-ium] dichloride hexahydrate Crystal data

**Table:**

C_26_H_22_N_6_^2+^·2(Cl^−^)·6(H_2_O)	*F*(000) = 1256
*M**_r_* = 597.49	*D*_x_ = 1.382 Mg m^−^^3^
Monoclinic, *P*2_1_/*n*	Mo *K*α radiation, λ = 0.71073 Å
*a* = 9.0061 (7) Å	Cell parameters from 3978 reflections
*b* = 15.1053 (10) Å	θ = 2.9–27.3°
*c* = 21.2487 (14) Å	µ = 0.28 mm^−^^1^
β = 96.752 (7)°	*T* = 150 K
*V* = 2870.6 (3) Å^3^	Needle, yellow
*Z* = 4	0.38 × 0.09 × 0.05 mm

### 2,2'-(Ethane-1,2-diyl)bis[3-(pyridin-2-yl)imidazo[1,5-a]pyridin-2-ium] dichloride hexahydrate Data collection

**Table:**

New Gemini, Dual, Cu at home/near, Atlas diffractometer	6542 measured reflections
Radiation source: fine-focus sealed X-ray tube, Enhance (Mo) X-ray Source	6542 independent reflections
Graphite monochromator	3396 reflections with *I* > 2σ(*I*)
Detector resolution: 10.6426 pixels mm^-1^	θ_max_ = 28.8°, θ_min_ = 2.4°
ω scans	*h* = −9→12
Absorption correction: analytical (CrysAlisPro; Rigaku OD, 2026)	*k* = −20→20
*T*_min_ = 0.933, *T*_max_ = 0.980	*l* = −27→27

### 2,2'-(Ethane-1,2-diyl)bis[3-(pyridin-2-yl)imidazo[1,5-a]pyridin-2-ium] dichloride hexahydrate Refinement

**Table:**

Refinement on *F*^2^	Primary atom site location: dual
Least-squares matrix: full	Hydrogen site location: mixed
*R*[*F*^2^ > 2σ(*F*^2^)] = 0.051	H-atom parameters constrained
*wR*(*F*^2^) = 0.091	*w* = 1/[σ^2^(*F*_o_^2^) + (0.0354*P*)^2^] where *P* = (*F*_o_^2^ + 2*F*_c_^2^)/3
*S* = 0.86	(Δ/σ)_max_ < 0.001
6542 reflections	Δρ_max_ = 0.27 e Å^−^^3^
380 parameters	Δρ_min_ = −0.34 e Å^−^^3^
0 restraints	

### 2,2'-(Ethane-1,2-diyl)bis[3-(pyridin-2-yl)imidazo[1,5-a]pyridin-2-ium] dichloride hexahydrate Special details

**Table:**

Geometry. All esds (except the esd in the dihedral angle between two l.s. planes) are estimated using the full covariance matrix. The cell esds are taken into account individually in the estimation of esds in distances, angles and torsion angles; correlations between esds in cell parameters are only used when they are defined by crystal symmetry. An approximate (isotropic) treatment of cell esds is used for estimating esds involving l.s. planes.
Refinement. Refined as a 2-component twin.

### 2,2'-(Ethane-1,2-diyl)bis[3-(pyridin-2-yl)imidazo[1,5-a]pyridin-2-ium] dichloride hexahydrate Fractional atomic coordinates and isotropic or equivalent isotropic displacement parameters (Å^2^)

**Table:**

	*x*	*y*	*z*	*U*_iso_*/*U*_eq_	
Cl1	0.34159 (8)	0.44925 (5)	0.27864 (3)	0.02028 (19)	
Cl2	0.34229 (8)	0.12488 (5)	0.45445 (4)	0.02505 (19)	
O1	0.3969 (2)	0.69367 (14)	0.30924 (12)	0.0397 (7)	
H1A	0.391339	0.751196	0.309624	0.060*	
O2	0.3441 (3)	0.65200 (15)	0.43752 (12)	0.0498 (7)	
H2A	0.378637	0.665517	0.402141	0.075*	
H2B	0.341652	0.594725	0.441537	0.075*	
O3	0.6113 (3)	0.67544 (13)	0.51139 (11)	0.0349 (6)	
H3A	0.631277	0.728534	0.525820	0.052*	
H3B	0.522942	0.674898	0.489763	0.052*	
O4	0.3695 (3)	0.91568 (14)	0.44330 (11)	0.0399 (6)	
H4A	0.445418	0.902545	0.470926	0.060*	
H4B	0.361142	0.972962	0.445853	0.060*	
O5	0.4114 (2)	0.87522 (14)	0.32004 (10)	0.0254 (5)	
H5A	0.393442	0.894352	0.357044	0.038*	
H5B	0.341090	0.898619	0.293589	0.038*	
O6	0.3141 (2)	0.47281 (13)	0.42080 (10)	0.0274 (5)	
H6A	0.343403	0.426310	0.443087	0.041*	
H6B	0.326415	0.463805	0.381280	0.041*	
N1	0.7510 (2)	0.75581 (14)	0.31869 (11)	0.0137 (6)	
N2	0.7252 (2)	0.79911 (14)	0.18463 (11)	0.0117 (5)	
N3	0.9513 (2)	0.75222 (14)	0.18391 (11)	0.0136 (6)	
N4	1.0080 (2)	0.58519 (14)	0.25551 (10)	0.0115 (5)	
N5	0.8298 (2)	0.55106 (14)	0.31092 (10)	0.0102 (5)	
N6	0.6229 (2)	0.59715 (14)	0.19762 (11)	0.0154 (6)	
C1	0.7612 (3)	0.75961 (18)	0.38213 (15)	0.0208 (7)	
H1	0.677703	0.740553	0.402076	0.025*	
C2	0.8859 (3)	0.78960 (19)	0.42009 (14)	0.0211 (8)	
H2	0.888213	0.789370	0.464908	0.025*	
C3	1.0059 (3)	0.81963 (18)	0.39254 (14)	0.0215 (8)	
H3	1.093105	0.840312	0.417761	0.026*	
C4	0.9977 (3)	0.81926 (18)	0.32654 (13)	0.0173 (7)	
H4	1.077720	0.841562	0.305784	0.021*	
C5	0.8707 (3)	0.78580 (17)	0.29215 (13)	0.0116 (7)	
C6	0.8515 (3)	0.78033 (17)	0.22228 (13)	0.0121 (7)	
C7	0.5893 (3)	0.83128 (17)	0.20013 (14)	0.0161 (7)	
H7	0.575635	0.843549	0.242923	0.019*	
C8	0.4782 (3)	0.84457 (18)	0.15337 (14)	0.0212 (7)	
H8	0.385170	0.866297	0.163785	0.025*	
C9	0.4944 (3)	0.82734 (19)	0.08866 (15)	0.0234 (8)	
H9	0.413001	0.837278	0.056747	0.028*	
C10	0.6264 (3)	0.79669 (19)	0.07286 (14)	0.0214 (8)	
H10	0.638820	0.785341	0.029810	0.026*	
C11	0.7453 (3)	0.78182 (18)	0.12115 (13)	0.0150 (7)	
C12	0.8885 (3)	0.75273 (17)	0.12243 (14)	0.0150 (7)	
H12	0.936291	0.735764	0.086739	0.018*	
C13	1.1011 (3)	0.71386 (18)	0.20266 (14)	0.0162 (7)	
H13A	1.141404	0.736686	0.244920	0.019*	
H13B	1.169780	0.732209	0.171945	0.019*	
C14	1.0936 (3)	0.61436 (17)	0.20481 (13)	0.0134 (6)	
H14A	1.045755	0.591735	0.163619	0.016*	
H14B	1.196154	0.589822	0.212102	0.016*	
C15	0.8620 (3)	0.56256 (16)	0.25123 (13)	0.0101 (6)	
C16	0.6964 (3)	0.52366 (17)	0.33213 (14)	0.0149 (7)	
H16	0.610915	0.510540	0.303019	0.018*	
C17	0.6923 (3)	0.51638 (18)	0.39477 (14)	0.0212 (7)	
H17	0.601920	0.498198	0.409892	0.025*	
C18	0.8192 (3)	0.53505 (18)	0.43937 (14)	0.0200 (7)	
H18	0.811986	0.530007	0.483475	0.024*	
C19	0.9489 (3)	0.55973 (18)	0.41937 (13)	0.0169 (7)	
H19	1.033991	0.571571	0.448994	0.020*	
C20	0.9570 (3)	0.56788 (17)	0.35347 (13)	0.0129 (7)	
C21	1.0676 (3)	0.58904 (17)	0.31752 (13)	0.0132 (7)	
H21	1.167919	0.603788	0.332841	0.016*	
C22	0.7543 (3)	0.55631 (17)	0.19346 (12)	0.0115 (6)	
C23	0.7883 (3)	0.51304 (17)	0.13998 (13)	0.0159 (7)	
H23	0.881120	0.483198	0.139762	0.019*	
C24	0.6847 (3)	0.51395 (19)	0.08661 (14)	0.0230 (8)	
H24	0.705556	0.486108	0.048541	0.028*	
C25	0.5512 (3)	0.5559 (2)	0.08991 (14)	0.0253 (8)	
H25	0.478077	0.558064	0.053876	0.030*	
C26	0.5239 (3)	0.59480 (18)	0.14577 (14)	0.0197 (7)	
H26	0.429119	0.621606	0.147604	0.024*	
H1B	0.494800	0.679000	0.299700	0.030*	

### 2,2'-(Ethane-1,2-diyl)bis[3-(pyridin-2-yl)imidazo[1,5-a]pyridin-2-ium] dichloride hexahydrate Atomic displacement parameters (Å^2^)

**Table:**

	*U*^11^	*U*^22^	*U*^33^	*U*^12^	*U*^13^	*U*^23^
Cl1	0.0174 (4)	0.0227 (4)	0.0209 (4)	−0.0004 (3)	0.0030 (3)	−0.0044 (3)
Cl2	0.0263 (5)	0.0283 (4)	0.0213 (4)	−0.0060 (4)	0.0059 (4)	−0.0017 (4)
O1	0.0234 (14)	0.0268 (13)	0.0667 (18)	0.0002 (11)	−0.0040 (13)	0.0101 (13)
O2	0.0586 (18)	0.0339 (15)	0.0542 (18)	−0.0039 (14)	−0.0043 (15)	0.0069 (12)
O3	0.0443 (17)	0.0280 (13)	0.0326 (15)	0.0009 (12)	0.0061 (12)	−0.0006 (11)
O4	0.0471 (18)	0.0340 (14)	0.0351 (15)	0.0009 (12)	−0.0100 (13)	−0.0082 (11)
O5	0.0195 (13)	0.0286 (12)	0.0269 (13)	0.0043 (10)	−0.0021 (10)	−0.0017 (11)
O6	0.0337 (14)	0.0309 (13)	0.0184 (12)	−0.0023 (11)	0.0059 (11)	0.0035 (10)
N1	0.0145 (15)	0.0156 (13)	0.0127 (14)	0.0017 (10)	0.0081 (12)	0.0011 (11)
N2	0.0109 (14)	0.0112 (13)	0.0139 (14)	0.0023 (10)	0.0052 (12)	0.0012 (11)
N3	0.0104 (14)	0.0138 (13)	0.0176 (15)	0.0028 (10)	0.0061 (12)	0.0018 (11)
N4	0.0087 (14)	0.0147 (13)	0.0114 (13)	0.0034 (10)	0.0023 (11)	0.0011 (10)
N5	0.0079 (13)	0.0127 (12)	0.0103 (13)	−0.0016 (11)	0.0020 (11)	0.0000 (11)
N6	0.0088 (14)	0.0169 (13)	0.0208 (14)	0.0005 (11)	0.0025 (11)	−0.0003 (11)
C1	0.0193 (19)	0.0221 (18)	0.0225 (19)	0.0014 (14)	0.0089 (16)	0.0002 (14)
C2	0.026 (2)	0.0214 (18)	0.0174 (18)	0.0023 (15)	0.0067 (16)	−0.0010 (14)
C3	0.0220 (19)	0.0204 (18)	0.0219 (19)	−0.0044 (14)	0.0020 (15)	−0.0025 (14)
C4	0.0153 (18)	0.0197 (17)	0.0183 (18)	−0.0031 (13)	0.0075 (14)	0.0012 (14)
C5	0.0103 (17)	0.0092 (14)	0.0161 (17)	0.0048 (12)	0.0044 (13)	−0.0006 (13)
C6	0.0124 (17)	0.0076 (14)	0.0171 (17)	−0.0015 (12)	0.0049 (14)	0.0016 (13)
C7	0.0107 (17)	0.0148 (16)	0.0248 (18)	0.0022 (13)	0.0110 (15)	0.0023 (14)
C8	0.0151 (18)	0.0177 (17)	0.0309 (19)	0.0050 (13)	0.0038 (16)	0.0054 (14)
C9	0.0200 (19)	0.0222 (18)	0.0264 (19)	0.0052 (14)	−0.0039 (15)	0.0065 (15)
C10	0.028 (2)	0.0209 (18)	0.0159 (18)	0.0040 (15)	0.0059 (16)	0.0064 (14)
C11	0.0174 (18)	0.0147 (16)	0.0143 (17)	0.0017 (13)	0.0075 (14)	0.0040 (13)
C12	0.0140 (18)	0.0169 (17)	0.0146 (18)	0.0005 (13)	0.0043 (14)	0.0016 (13)
C13	0.0069 (17)	0.0240 (17)	0.0179 (17)	0.0013 (13)	0.0026 (13)	0.0036 (14)
C14	0.0049 (15)	0.0236 (17)	0.0120 (15)	−0.0006 (13)	0.0021 (12)	−0.0001 (14)
C15	0.0083 (16)	0.0077 (14)	0.0148 (16)	0.0022 (12)	0.0035 (13)	0.0005 (13)
C16	0.0092 (16)	0.0150 (15)	0.0215 (18)	0.0014 (13)	0.0052 (14)	0.0045 (13)
C17	0.0175 (18)	0.0211 (17)	0.028 (2)	0.0068 (14)	0.0169 (16)	0.0109 (15)
C18	0.0234 (19)	0.0227 (18)	0.0152 (17)	0.0096 (14)	0.0076 (15)	0.0060 (14)
C19	0.0203 (19)	0.0207 (17)	0.0095 (16)	0.0059 (14)	0.0007 (14)	0.0000 (13)
C20	0.0112 (17)	0.0128 (16)	0.0146 (16)	0.0051 (12)	0.0013 (13)	0.0009 (13)
C21	0.0080 (16)	0.0157 (16)	0.0144 (16)	0.0009 (12)	−0.0045 (14)	0.0018 (12)
C22	0.0107 (16)	0.0087 (14)	0.0151 (16)	−0.0024 (13)	0.0008 (13)	0.0015 (13)
C23	0.0102 (16)	0.0159 (16)	0.0215 (18)	0.0015 (13)	0.0020 (14)	−0.0026 (14)
C24	0.027 (2)	0.0253 (18)	0.0159 (18)	−0.0056 (15)	−0.0002 (15)	−0.0093 (14)
C25	0.023 (2)	0.0314 (19)	0.0185 (18)	−0.0032 (16)	−0.0101 (15)	−0.0011 (16)
C26	0.0077 (16)	0.0232 (18)	0.0264 (19)	−0.0035 (13)	−0.0055 (14)	0.0035 (14)

### 2,2'-(Ethane-1,2-diyl)bis[3-(pyridin-2-yl)imidazo[1,5-a]pyridin-2-ium] dichloride hexahydrate Geometric parameters (Å, º)

**Table:**

O1—H1A	0.8705	C5—C6	1.477 (4)
O1—H1B	0.954 (2)	C7—H7	0.9500
O2—H2A	0.8710	C7—C8	1.340 (4)
O2—H2B	0.8699	C8—H8	0.9500
O3—H3A	0.8698	C8—C9	1.423 (4)
O3—H3B	0.8708	C9—H9	0.9500
O4—H4A	0.8702	C9—C10	1.355 (4)
O4—H4B	0.8707	C10—H10	0.9500
O5—H5A	0.8705	C10—C11	1.411 (4)
O5—H5B	0.8706	C11—C12	1.360 (4)
O6—H6A	0.8706	C12—H12	0.9500
O6—H6B	0.8707	C13—H13A	0.9900
N1—C1	1.342 (4)	C13—H13B	0.9900
N1—C5	1.352 (3)	C13—C14	1.505 (4)
N2—C6	1.342 (3)	C14—H14A	0.9900
N2—C7	1.392 (3)	C14—H14B	0.9900
N2—C11	1.407 (3)	C15—C22	1.475 (4)
N3—C6	1.351 (3)	C16—H16	0.9500
N3—C12	1.361 (3)	C16—C17	1.340 (4)
N3—C13	1.479 (3)	C17—H17	0.9500
N4—C14	1.464 (3)	C17—C18	1.425 (4)
N4—C15	1.352 (3)	C18—H18	0.9500
N4—C21	1.364 (3)	C18—C19	1.342 (4)
N5—C15	1.345 (3)	C19—H19	0.9500
N5—C16	1.395 (3)	C19—C20	1.416 (4)
N5—C20	1.397 (3)	C20—C21	1.363 (4)
N6—C22	1.346 (3)	C21—H21	0.9500
N6—C26	1.334 (3)	C22—C23	1.377 (4)
C1—H1	0.9500	C23—H23	0.9500
C1—C2	1.380 (4)	C23—C24	1.382 (4)
C2—H2	0.9500	C24—H24	0.9500
C2—C3	1.366 (4)	C24—C25	1.368 (4)
C3—H3	0.9500	C25—H25	0.9500
C3—C4	1.396 (4)	C25—C26	1.372 (4)
C4—H4	0.9500	C26—H26	0.9500
C4—C5	1.378 (4)		
			
H1A—O1—H1B	106.8	C12—C11—C10	134.7 (3)
H2A—O2—H2B	109.5	N3—C12—H12	126.1
H3A—O3—H3B	109.5	C11—C12—N3	107.9 (3)
H4A—O4—H4B	104.6	C11—C12—H12	126.1
H5A—O5—H5B	104.5	N3—C13—H13A	109.5
H6A—O6—H6B	109.5	N3—C13—H13B	109.5
C1—N1—C5	116.2 (2)	N3—C13—C14	110.9 (2)
C6—N2—C7	129.8 (2)	H13A—C13—H13B	108.0
C6—N2—C11	109.7 (2)	C14—C13—H13A	109.5
C7—N2—C11	120.5 (2)	C14—C13—H13B	109.5
C6—N3—C12	110.3 (2)	N4—C14—C13	110.6 (2)
C6—N3—C13	127.7 (2)	N4—C14—H14A	109.5
C12—N3—C13	121.6 (2)	N4—C14—H14B	109.5
C15—N4—C14	128.2 (2)	C13—C14—H14A	109.5
C15—N4—C21	110.2 (2)	C13—C14—H14B	109.5
C21—N4—C14	121.1 (2)	H14A—C14—H14B	108.1
C15—N5—C16	129.2 (2)	N4—C15—C22	127.7 (2)
C15—N5—C20	109.6 (2)	N5—C15—N4	106.6 (2)
C16—N5—C20	121.1 (2)	N5—C15—C22	125.7 (2)
C26—N6—C22	116.1 (2)	N5—C16—H16	121.0
N1—C1—H1	118.1	C17—C16—N5	118.1 (3)
N1—C1—C2	123.8 (3)	C17—C16—H16	121.0
C2—C1—H1	118.1	C16—C17—H17	119.0
C1—C2—H2	120.4	C16—C17—C18	122.1 (3)
C3—C2—C1	119.3 (3)	C18—C17—H17	119.0
C3—C2—H2	120.4	C17—C18—H18	119.9
C2—C3—H3	120.7	C19—C18—C17	120.3 (3)
C2—C3—C4	118.6 (3)	C19—C18—H18	119.9
C4—C3—H3	120.7	C18—C19—H19	120.5
C3—C4—H4	120.8	C18—C19—C20	119.1 (3)
C5—C4—C3	118.5 (3)	C20—C19—H19	120.5
C5—C4—H4	120.8	N5—C20—C19	119.4 (2)
N1—C5—C4	123.6 (3)	C21—C20—N5	106.1 (2)
N1—C5—C6	113.3 (2)	C21—C20—C19	134.5 (3)
C4—C5—C6	123.1 (3)	N4—C21—H21	126.2
N2—C6—N3	106.5 (2)	C20—C21—N4	107.5 (2)
N2—C6—C5	125.4 (2)	C20—C21—H21	126.2
N3—C6—C5	128.1 (3)	N6—C22—C15	114.4 (2)
N2—C7—H7	120.7	N6—C22—C23	123.7 (3)
C8—C7—N2	118.5 (3)	C23—C22—C15	121.9 (2)
C8—C7—H7	120.7	C22—C23—H23	120.7
C7—C8—H8	118.8	C22—C23—C24	118.6 (3)
C7—C8—C9	122.5 (3)	C24—C23—H23	120.7
C9—C8—H8	118.8	C23—C24—H24	120.8
C8—C9—H9	120.2	C25—C24—C23	118.4 (3)
C10—C9—C8	119.6 (3)	C25—C24—H24	120.8
C10—C9—H9	120.2	C24—C25—H25	120.3
C9—C10—H10	120.4	C24—C25—C26	119.4 (3)
C9—C10—C11	119.2 (3)	C26—C25—H25	120.3
C11—C10—H10	120.4	N6—C26—C25	123.8 (3)
N2—C11—C10	119.7 (2)	N6—C26—H26	118.1
C12—C11—N2	105.7 (2)	C25—C26—H26	118.1
			
N1—C1—C2—C3	1.8 (4)	C11—N2—C7—C8	0.6 (4)
N1—C5—C6—N2	40.3 (4)	C12—N3—C6—N2	−0.3 (3)
N1—C5—C6—N3	−136.2 (3)	C12—N3—C6—C5	176.8 (3)
N2—C7—C8—C9	−0.2 (4)	C12—N3—C13—C14	−77.9 (3)
N2—C11—C12—N3	0.2 (3)	C13—N3—C6—N2	−172.3 (2)
N3—C13—C14—N4	−66.3 (3)	C13—N3—C6—C5	4.8 (4)
N4—C15—C22—N6	−132.1 (3)	C13—N3—C12—C11	172.6 (2)
N4—C15—C22—C23	47.1 (4)	C14—N4—C15—N5	−172.6 (2)
N5—C15—C22—N6	44.1 (4)	C14—N4—C15—C22	4.2 (4)
N5—C15—C22—C23	−136.8 (3)	C14—N4—C21—C20	172.9 (2)
N5—C16—C17—C18	−0.5 (4)	C15—N4—C14—C13	96.2 (3)
N5—C20—C21—N4	0.2 (3)	C15—N4—C21—C20	0.3 (3)
N6—C22—C23—C24	2.4 (4)	C15—N5—C16—C17	179.4 (3)
C1—N1—C5—C4	−0.3 (4)	C15—N5—C20—C19	−180.0 (2)
C1—N1—C5—C6	−179.6 (2)	C15—N5—C20—C21	−0.6 (3)
C1—C2—C3—C4	0.3 (4)	C15—C22—C23—C24	−176.6 (3)
C2—C3—C4—C5	−2.2 (4)	C16—N5—C15—N4	−176.9 (2)
C3—C4—C5—N1	2.3 (4)	C16—N5—C15—C22	6.3 (4)
C3—C4—C5—C6	−178.6 (3)	C16—N5—C20—C19	−2.1 (4)
C4—C5—C6—N2	−138.9 (3)	C16—N5—C20—C21	177.3 (2)
C4—C5—C6—N3	44.5 (4)	C16—C17—C18—C19	−0.9 (4)
C5—N1—C1—C2	−1.7 (4)	C17—C18—C19—C20	0.7 (4)
C6—N2—C7—C8	−179.8 (3)	C18—C19—C20—N5	0.7 (4)
C6—N2—C11—C10	179.9 (2)	C18—C19—C20—C21	−178.4 (3)
C6—N2—C11—C12	−0.4 (3)	C19—C20—C21—N4	179.4 (3)
C6—N3—C12—C11	0.1 (3)	C20—N5—C15—N4	0.8 (3)
C6—N3—C13—C14	93.3 (3)	C20—N5—C15—C22	−176.0 (2)
C7—N2—C6—N3	−179.2 (2)	C20—N5—C16—C17	1.9 (4)
C7—N2—C6—C5	3.6 (4)	C21—N4—C14—C13	−74.9 (3)
C7—N2—C11—C10	−0.4 (4)	C21—N4—C15—N5	−0.7 (3)
C7—N2—C11—C12	179.3 (2)	C21—N4—C15—C22	176.0 (2)
C7—C8—C9—C10	−0.3 (4)	C22—N6—C26—C25	−1.7 (4)
C8—C9—C10—C11	0.5 (4)	C22—C23—C24—C25	−1.7 (4)
C9—C10—C11—N2	−0.1 (4)	C23—C24—C25—C26	−0.6 (4)
C9—C10—C11—C12	−179.7 (3)	C24—C25—C26—N6	2.4 (5)
C10—C11—C12—N3	179.8 (3)	C26—N6—C22—C15	178.4 (2)
C11—N2—C6—N3	0.5 (3)	C26—N6—C22—C23	−0.7 (4)
C11—N2—C6—C5	−176.7 (3)		

### 2,2'-(Ethane-1,2-diyl)bis[3-(pyridin-2-yl)imidazo[1,5-a]pyridin-2-ium] dichloride hexahydrate Hydrogen-bond geometry (Å, º)

**Table:**

*D*—H···*A*	*D*—H	H···*A*	*D*···*A*	*D*—H···*A*
O1—H1*A*···O5	0.87	1.89	2.754 (3)	170
O2—H2*A*···O1	0.87	2.04	2.891 (4)	164
O2—H2*B*···O6	0.87	1.90	2.739 (3)	161
O3—H3*A*···Cl2^i^	0.87	2.26	3.119 (2)	169
O3—H3*B*···O2	0.87	1.88	2.737 (4)	169
O5—H5*A*···O4	0.87	1.90	2.758 (3)	169
O5—H5*B*···Cl1^ii^	0.87	2.25	3.117 (2)	176
O6—H6*A*···O3^i^	0.87	1.84	2.705 (3)	175
O6—H6*B*···Cl1	0.87	2.21	3.081 (2)	175

Symmetry codes: (i) −*x*+1, −*y*+1, −*z*+1; (ii) −*x*+1/2, *y*+1/2, −*z*+1/2.
